# Development of a clinical decision support tool for diagnostic imaging use in patients with low back pain: a study protocol

**DOI:** 10.1186/s41512-019-0047-8

**Published:** 2019-01-14

**Authors:** Jill A. Hayden, Rachel Ogilvie, Samuel Alan Stewart, Simon French, Samuel Campbell, Kirk Magee, Patrick Slipp, George Wells, Ian Stiell

**Affiliations:** 1 Department Community Health and Epidemiology, Dalhousie University, 5790 University Avenue, Halifax, NS B3H 1V7 Canada; 2 School of Rehabilitation Therapy, Queen’s University, Louise D. Acton Building, 31 George St, Kingston, ON K7L 3N6 Canada; 3 Department of Chiropractic, Macquarie University, Sydney, NSW 2109 Australia; 4 Department of Emergency Medicine, Dalhousie University, Emergency Medicine, Nova Scotia Health Authority, QEII Health Sciences Centre, Suite 355, 1796 Summer Street, Halifax, NS B3H 3A7 Canada; 5 Department of Radiology, Nova Scotia Health Authority, QEII Health Sciences Centre, Suite 355, 1796 Summer Street, Halifax, NS B3H 3A7 Canada; 6 Epidemiology and Community Medicine, University of Ottawa, Cardiovascular Research Methods Centre, University of Ottawa Heart Institute, 40 Ruskin Street, Ottawa, ON K1Y 4W7 Canada; 7 Department of Emergency Medicine, Ottawa Hospital Research Institute, University of Ottawa, 1053 Carling Avenue, Ottawa, ON K1Y 4E9 Canada

**Keywords:** Low back pain, Diagnostic imaging, Red flags, Clinical decision support tool, Prediction model, Knowledge translation

## Abstract

**Background:**

Low back pain is one of the most common and disabling health problems in Canada and internationally. In most cases, low back pain is a benign, self-limiting condition that can be managed with little diagnostic investigation or treatment. Yet contrary to clinical practice guideline recommendations, diagnostic imaging (here meaning X-ray, MRI, CT) is commonly used in the assessment of low back pain. Diagnostic imaging is of limited value in most cases, exposing patients to unnecessary radiation and leading to increased health services use and worse patient health outcomes. The Choosing Wisely campaign has highlighted the need to reduce diagnostic imaging for low back pain; however, no clinical decision rules are available.

**Methods:**

This project will develop a clinical decision support tool for appropriate use of diagnostic imaging for patients with low back pain in the emergency department. We will conduct a prospective cohort study at five Canadian emergency departments. The study will follow recommendations for prediction model development and testing. The study population will be 4000 patients presenting to the emergency department with low back pain. We will assess potential clinical indications of emergent-cause (i.e., “red flag” items), including clinical characteristics and past history. Our outcome, emergent-cause for low back pain such as fracture, cancer, infection, or cauda equina syndrome, will be assessed at discharge and at 1-, 3-, and 12-month follow-up periods using information from self-report and health administrative data. We will construct and assess the performance of a multivariable prediction model that has strong measurement properties, presented as a clinical decision support tool acceptable to knowledge users.

**Discussion:**

Practice guidelines describe “red flags” for which diagnostic imaging is likely appropriate. However, recommendations across guidelines are discordant, and few studies have evaluated these criteria to determine which characteristics best predict emergent etiology that warrant diagnostic imaging. A clinical decision support tool, that recommends diagnostic imaging where appropriate, has the potential to improve clinical care and patient outcomes and reduce costs associated with managing low back pain patients.

**Electronic supplementary material:**

The online version of this article (10.1186/s41512-019-0047-8) contains supplementary material, which is available to authorized users.

## Background

Low back pain is common and costly. Almost every Canadian suffers from low back pain at some point in their lives [1]. It is a leading cause of disability nationally and globally (the number one cause in 2015 [2]) and results in enormous direct health care and lost productivity costs [3–6]. In Canada, it is one of the most common reasons for seeking health care: back pain is the third most common presenting complaint at emergency departments for individuals aged 20–64 years [7]. There are approximately 360,000 visits to Canadian emergency departments for low back pain each year.

In most cases, low back pain is non-specific, that is, it is a benign, self-limiting condition that can be managed with little diagnostic investigation or treatment. Numerous systematic reviews and practice guidelines support the limited use of diagnostic imaging for the investigation and treatment of non-specific low back pain [8–11], recommending plain-film X-ray, MRI, or CT only in special circumstances for patients with low back pain when there are “red flag” indications [8]. Red flags are clinical characteristics suggestive of underlying emergent cause, including potential fracture, cancer, infection, or cauda equina syndrome. These diagnoses are rare, occurring in less than 10% of low back pain cases [12, 13].

Overuse of diagnostic imaging for low back pain (here meaning X-ray, MRI, CT) results in wasted resources and harms. Contrary to recommendations from clinical practice guidelines, diagnostic imaging for non-specific low back pain remains high [14, 15]. A recent study by our team in a Nova Scotia emergency department found that 30% of patients presenting to the emergency department, diagnosed with non-specific low back pain, had received diagnostic imaging [16], much higher than the expected rate of 10%. Emery et al. reported that only 44% of requests for lumbar spine MRI were considered appropriate [17]. Harms of inappropriate diagnostic imaging include exposing patients to unnecessary radiation, with lumbar spine X-ray having an exposure 75 times higher than that of a chest X-ray [18]. Furthermore, diagnostic imaging for low back pain has been shown to result in more subsequent testing and increased use of health services [19], including surgery [20], with no improvement in patient outcomes. This is potentially related to high false positive rates for several recommended red flags [21, 22] and “coincidental” radiological findings [23, 24]. Importantly, patients with non-specific low back pain who receive diagnostic imaging have worse long-term health outcomes [25, 26]. While the overuse of diagnostic imaging is recognized as problematic, there are also potential harms, for example, a delayed emergent diagnosis and management that may be associated with not using diagnostic imaging when appropriate.

Clinical prediction models, or clinical decision support tools, can improve practice and patient outcomes. Clinical support tools, built from multivariable prediction models, statistically combine information about patient and disease characteristics to assist providers in making patient-specific and evidence-informed decisions at the point of care. They can be used to shift practice patterns and to educate providers and patients. Clinical decision support tools have been used successfully in many fields [27, 28], including informing the appropriate use of radiographs for ankle sprain (Ottawa Ankle Rules [29–31]), and the necessity for immobilization and diagnostic imaging in trauma patients with neck pain (Canadian C-Spine Rule [32, 33]). The Ottawa Ankle Rules have led to decreased use of ankle radiography, wait times, and healthcare costs [34].

Few studies have adequately investigated the use of red flags as diagnostic criteria for emergent-cause low back pain. There are discordant recommendations for diagnostic imaging across clinical practice guidelines, high false positive rates reported for several recommended red flags [21], and uncertainty about how characteristics should be combined to inform decision-making. Although diagnostic accuracy of red flags for emergent disease in conditions such as low back pain is a challenging research topic due to small event rate, particularly in primary care, the evaluation of the performance of combinations of red flags is both necessary and promising [35]. Two earlier studies have attempted to develop decision tools for low back pain [21, 36]. These studies were conducted in primary care settings, limited by insufficient sample size, and a low number of emergent cases were identified. Moreover, they did not examine all emergent causes of low back pain that may warrant diagnostic imaging, instead only focusing on vertebral fractures.

The primary objective of this study is to develop a clinical decision support tool for the appropriate use of diagnostic imaging for patients with low back pain in the emergency department setting. The tool, once externally validated in a future study, will stratify patients by risk and accurately predict those patients presenting to the emergency department who are likely to have an emergent cause for their low back pain and therefore require imaging. Secondary study objectives are to provide prevalence estimates of low back pain with emergent-cause in the emergency department setting, and predictive accuracy data about currently recommended individual red flags and combinations of red flag screening items that should be recommended in clinical guidelines.

## Methods

This cohort study of patients who present to the emergency department with a primary complaint of low back pain will prospectively collect primary data, develop and test a prediction model, and develop a clinical decision support tool through established methods [37–39]. We will select potential variables, develop the prediction model, and assess the model performance (overall goodness-of-fit, calibration, discrimination, and clinical usefulness). We will present the clinical decision support tool as a simple index. Future studies will test external validity and include impact assessment.

We will follow guidelines for the development of clinical decision support tools [40–42] and the Transparent Reporting of a multivariable prediction model for Individual Prognosis or Diagnosis (TRIPOD) checklist [43].

### Study sites and participants

Data for this study will be collected from three urban tertiary care centers and two community emergency medicine sites located in Nova Scotia and Ontario, Canada, all of which provide 24-h, 7 days/week care. The studied emergency departments vary by density and patient volume, university affiliation, and the presence of a rapid assessment unit. Data collection from five different research-oriented locations will enhance geographic generalizability while ensuring feasibility. To assess generalizability, we will compare basic demographic characteristics of our eligible study population with that of the eligible population from the Canadian Institute for Health Information, National Ambulatory Care Reporting System, which represents approximately 63% of all emergency department visits in Canada.

The target population for our study is adults who present to the emergency department for low back pain. The sample will include all patients aged 18 and over with a valid provincial health plan number who present to one of the study sites’ emergency departments during the data collection period from approximately January 2019 to July 2020 with a primary complaint of low back pain (emergency department intake complaint codes: “back pain” or “traumatic back/spine injury”; we have previously observed both of these triage codes used for non-specific low back pain diagnoses); pain in the region bound by T12 and the lower gluteal fold, with or without pain referred beyond this region. We will exclude patients who are pregnant, those unable to understand English, and those with severe cognitive impairment that would negate consent.

The target recruitment for participants in our study is 4000 consecutive eligible patients, enrolled over a period of 15–18 months (approximately 50% from each province). Electronic notification at registration will alert triage staff to potentially eligible patients. Triage staff will subsequently give the patient the participant self-report survey and insert a physician data form into the patient chart for completion by the treating physician. Study procedures have been developed to optimize patient recruitment and minimize missing data, including integration of recruitment and data collection with the care process, raising healthcare team awareness of the study, training, and monitoring.

### Candidate predictor variables

We will assess potential red flag characteristics suggestive of cancer, fracture, infection, or cauda equina syndrome, which are all emergent causes that may present as low back pain and warrant diagnostic imaging. We will consider as candidate predictor variable characteristics that have evidence of predictive accuracy (positive likelihood ratio > 5.0), are recommended by the American College of Physicians clinical practice guideline [9] or the American College of Radiology [44], and/or identified by clinical experts. Several recent systematic reviews and studies have guided our selection and measurement of potential items [13, 22, 45]. Potential items, specific to at least one of the four emergent diagnoses investigated, will include age, sex, medical history, clinical characteristics, and the results of immediately available tests (Additional file 1).

### Data sources and collection

**Fig. 1:**
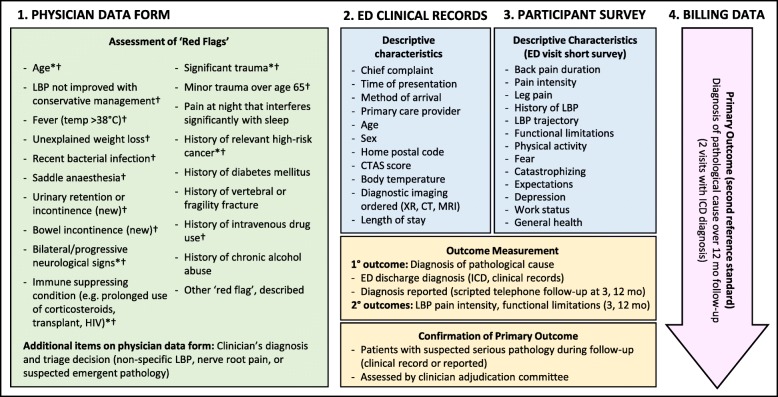
Baseline variables, including red flags and descriptive characteristics, and outcome variables of four data sources. The asterisk indicates the red flag with evidence of positive likelihood ratio > 5.0; the dagger represents the red flag described in the American College of Physicians low back pain clinical practice guideline [9] or by the American College of Radiology [8]; LBP, low back pain; CTAS, Canadian Triage and Acuity Score; ICD, International Classification of Disease; XR, plain-film X-ray; CT, computed tomography; MRI, magnetic resonance imaging; ED, emergency department

To develop a prediction model, we will collect participant characteristics (potential red flags and descriptive characteristics at the time of presentation to the emergency department (index visit)) and health outcomes (reference standard assessed over a 12-month follow-up period). To do this, we will use data from four sources: (1) physician data form (index visit), (2) emergency department clinical records (index visit), (3) participant surveys (index visit and follow-up), and (4) provincial administrative health “billing” data (follow-up) (Fig. 1).

#### Data collection at index visit (potential red flags and descriptive characteristics)

We will collect participant information using a simple healthcare provider survey (physician data form), emergency department clinical records, and a participant survey. The physician data form will be a single page, self-administered hard copy in patients’ charts and will collect data about the patient’s red flag characteristics as well as an item asking for the attending physician’s assessment of the likelihood that the patient’s low back pain has an emergent cause. Physicians will be trained to complete the standardized assessment prior to the start of the study. We will conduct a blinded inter-rater reliability testing of items in a subset of 100 participants assessed by two independent physicians (sample adequate for agreement ≥ 0.5; 0.2 relative error).

Emergency department clinical record data for all eligible participants will be retrieved from the Emergency Department Information System, used by all study emergency departments, and from related medical records (charts). We will use a pre-tested MS Access database to obtain Emergency Department Information System data and confirm and complete as necessary with patient clinical records. These data will enable us to characterize the participant population (“Descriptive Characteristics”, Fig. 1). The participant self-report survey will be completed in the emergency department during the index visit. The survey will collect information about baseline characteristics unavailable in the Emergency Department Information System (including back pain history and episode/health characteristics) for complete description of the study population and to allow comparison with other low back pain cohorts (Additional file 2).

#### Data collection at follow-up

Our primary outcome is confirmed by the diagnosis of emergent-cause low back pain, which is a composite outcome defined as a confirmed diagnosis of fracture, cancer, infection, or cauda equina syndrome. It is clinically relevant to assess this composite outcome since all component diagnoses are equally important to the emergency medicine clinician to identify a patient who has a cause for their low back pain that requires different management. We expect that fracture will be the most common component diagnosis (approximately 70% of emergent-cause low back pain diagnoses).

We will determine the presence of emergent-cause low back pain (fracture, cancer, infection, or cauda equina syndrome) in two ways to provide robustness to our results: (1) using combined information from emergency department clinical records and participant self-report, and (2) through provincial administrative health data, each with 12 months of follow-up (Additional file 3).

First, we will collect information about physician-assigned diagnosis at emergency department discharge and any records of return visits from emergency department clinical records and from assessor-blinded participant self-report survey administered by telephone at 3 and 12 months. For participant follow-up, we will use a computerized tracking system with service and support from the Canadian Longitudinal Study of Health and Aging (Halifax), which has a computer-assisted telephone interview site. Following Henschke et al. [21], participants will be asked, “Low back pain is occasionally the result of a fracture, infection or cancer. Has a healthcare provider said that your back pain is caused by one of these rare diseases?” and prompted for additional details about their diagnosis and investigations received. Multiple points of follow-up will limit recall bias, and a 12-month follow-up will allow sufficient time for emergence of potential emergent causes of low back pain. All participants diagnosed or reporting a new diagnosis of emergent-cause low back pain will have their diagnosis confirmed through their patient record (if consent is granted) or be referred to a study doctor who will confirm their diagnosis.

Second, we will use provincial administrative health data (physician billings) to identify new diagnoses in the 12 months following the index visit that are likely associated with the low back pain episode. In Nova Scotia, Health Data Nova Scotia, and in Ontario, Institute for Clinical Evaluative Sciences, maintain copies of provincial medical services insurance databases, which capture all information on billing claims for health services provided by practicing physicians in the province, which are linked to individual participants’ provincial health card numbers. Relevant diagnoses associated with emergent causes of low back pain will be defined using diagnostic codes. We will explore the robustness of our results with this administrative reference standard. A strength of this secondary analysis is that it will allow us to test and refine a reliable administrative definition of emergent-cause low back pain diagnosis by comparing with available participant self-report survey data, facilitating future external validation studies in other settings.

#### Missing data

We will use three strategies to address potential for missing data: (1) study design and implementation methods to avoid missing data, (2) multiple sources of data to fill in missing data and to allow analyses to explore potential reasons and patterns of missing data, (3) describe missingness and testing assumptions about missingness to determine appropriate analyses, including complete case, and multiple imputation for key variables (red flags in the prediction model), with sensitivity analysis to test the impact of different assumptions about why data are missing for analyses.

All data collection instruments have been designed to reduce missingness in responses, both through the brevity of questions and the layout of the instruments. To ensure quality of data, we will train the emergency medicine physicians and other clinical staff about the study procedures, including patient and physician questionnaires. A challenge that we have anticipated is the completion of the physician checklist; we do not expect a substantial amount of missing data for individual red flag variables (since they are part of standard care), but rather fully non-completed physician checklists. Our methods will aim to limit this; however, in these cases, we do not expect that the reasons for missing red flag items will be related to the outcome (i.e., missing at random; limiting precision, but not introducing bias). In this situation, we will cautiously use a multiple imputation approach and compare these results to results in a complete case analysis. If multiple imputation and complete case analyses give different results, we will explore potential reasons for this [46].

### Data analysis

We will follow steps recommended by Stiell and Wells for the development of a clinical decision/prediction rule [40], further informed by literature from the field of prediction model development [37, 42, 47] and clinical decision support systems [48]. Our purpose is to develop an accurate and clinically useful multivariable model that can inform a clinical decision support tool.

#### Item selection

Our preliminary selection of items for the model has been guided by available systematic reviews and clinical practice guidelines. We will prioritize variables that are easy to collect, have good face validity and clinical acceptance, and acceptable inter-rater reliability for inclusion in the model. To judge the most promising variables, we will consider univariate associations, correlation between variables, extent and patterns of missing data, inter-rater reliability (K > 0.8), and clinically acceptable combinations of items. In model development, we will consider continuous variables (as recommended to not lose information). We do not plan a priori to examine interaction terms.

#### Model estimation

We will build the prediction model, accounting for non-independence of data from our five study sites, by constructing a multivariable logistic regression model including the most promising variables. We will compare models that are theoretically driven (a priori fully specified) to those generated using backward selection (simplified models). We will cautiously estimate model coefficients using bootstrap sampling and lasso penalized regression methods. The final prediction model may be modified based on findings from the internal validation. We aim to develop a model that has strong measurement properties, that it is sensitive and sufficiently specific, and is clinically acceptable, logical, and easy to use.

#### Assess model performance

We will assess the overall performance of the resulting prediction models by calculating discrimination (c-index and receiver operating curves) and calibration (predicted vs. observed plots) [42, 49]. We will assess the ability of the models to classify patients into risk groups across a range of thresholds: less than 1%, 1 to < 5%, 5 to < 10%, and greater than 10%. We will quantify the clinical usefulness of the prediction models using the net benefit approach to balance benefits and harms, proposed by Vickers at al. [50].

All statistical analyses will be conducted using Stata and R.

### Study sample size

The target sample of 4000 patients was calculated to have appropriate statistical power based on 10 events per variable for the outcome of interest, emergent-cause low back pain [42, 47], assuming 3–6% of participants experience the outcome event [35, 51, 52], and an analysis of 10 independent variables in our prediction model, accounting for 20% attrition at 12 months. Our expected 128 events will provide adequate sample size for internal validation [53].

### Clinical decision support tool presentation

The final step of model development will be translation into a practical risk stratification approach or tool for potential use by healthcare providers [54]. We will develop the clinical decision support tool from the prediction model, simplifying the regression coefficients to an easily calculated score. The practical tool will be presented along with expected probabilities of outcome and recommendations for diagnostic imaging and with sensitivity/specificity for low- versus high-risk groups. We will incorporate design considerations raised in the clinical decision support literature, in our team’s on-going related studies, and in stakeholder consultations.

## Discussion

Our methodological approaches will mitigate challenges specific to studying emergent-cause low back pain and in general for the development of prediction models/tools. Our prospective cohort design will allow inclusion of relevant predictors (credible, reliable, and measured appropriately) and limit missing values. We will include a large sample from a clinically relevant setting with high outcome prevalence to ensure sufficient sample size for adequate model development. Selection and attrition biases common to prospective studies will be mitigated using established recruitment protocols and support from emergency departments’ administration and staff for consecutive participation and expertise from the Halifax Canadian Longitudinal Study of Health and Aging site for complete follow-up; potential participation bias will be assessed using administrative data. A limitation of our study includes the lack of a single standard approach to identify our target condition (emergent-cause low back pain); however, we will use multiple approaches and compare physician diagnosis/patient self-report, with an administrative data defined measure.

This study will provide much needed evidence on the predictive value of individuals and combined red flags and will produce an internally valid, reliable, and acceptable clinical decision support tool to inform appropriate decisions on diagnostic imaging for patients presenting with low back pain (note that the tool is not intended to support decisions about which diagnostic imaging approach is indicated). Future research will test external validity of the tool in other settings, evaluate implementation, and study the impact of the tool on patient and health services outcomes; this is planned by our team in collaboration with national and international networks. The clear need for a clinical decision support tool and engagement of end-users throughout our study will ensure its usefulness and positive impact on clinical practice.

This project will result in new knowledge about the prevalence of emergent-cause of low back pain and the predictive accuracy of clinical characteristics to inform appropriate diagnostic imaging. The resulting clinical decision support tool will establish a foundation to improve the delivery of appropriate care for patients by enabling evidence-based decision-making, while seizing the opportunity to reduce unnecessary costs and harms and improving patient outcomes for this common health condition.

## Additional files

Additional file 1: Physician report questionnaire items. (PDF 76 kb)

Additional file 2: Participant self-report questionnaire. (PDF 204 kb)

Additional file 3: ICD codes to identify emergent-cause low back pain. (PDF 14 kb)

## Abbreviations

ED: Emergency department
TRIPOD: Transparent Reporting of a multivariable prediction model for Individual Prognosis or Diagnosis
