# Supplementary material for: Development of a clinical decision support tool for diagnostic imaging use in patients with low back pain: a study protocol
Source: Diagn Progn Res. 2019 Jan 14;3:1. doi: 10.1186/s41512-019-0047-8 (PMC6460553; doi:10.1186/s41512-019-0047-8)
Supplement: Supplementary file 1 — Physician report questionnaire items. (PDF 76 kb) [file 41512_2019_47_MOESM1_ESM.pdf]

Appendix 1: Items to be included in the physician report 'red flag' questionnaire.

**Physician Report Questionnaire Items**
Significant trauma
Minor trauma, age >65
Night pain that interferes significantly with sleep
Back pain that has not improved with conservative management (>4 weeks)
Unexplained weight loss
Fever (self-report)
Bilateral or progressive neurological signs
Urinary retention or incontinence
Bowel incontinence
History of vertebral or fragility fracture
History of relevant high-risk cancer (e.g. prostate, breast, lung, kidney)
Recent relevant bacterial infection (e.g. UTI, skin infection, bacteremia)
Immune suppressing condition or medication
Diabetes mellitus
Current intravenous drug use
Chronic alcohol abuse
Other "red flag". *Please describe:*
Body temperature >38°C
Lower limb weakness
Absent reflexes in lower limbs
Saddle anaesthesia
Post void residual > 200 ml
Decreased or absent rectal tone
