# Supplementary material for: Development of a clinical decision support tool for diagnostic imaging use in patients with low back pain: a study protocol
Source: Diagn Progn Res. 2019 Jan 14;3:1. doi: 10.1186/s41512-019-0047-8 (PMC6460553; doi:10.1186/s41512-019-0047-8)
Supplement: Supplementary file 2 — Participant self-report questionnaire. (PDF 204 kb) [file 41512_2019_47_MOESM2_ESM.pdf]

Appendix 2: Participant self-report questionnaire completed at baseline.

Back pain history

1. How long have you had your **current episode** of low back pain? **(check one)**

☐ Less than 1 week
☐ 1 week to 1 month
☐ 1 to 3 months
☐ 3 to 12 months
☐ More than 12 months

2. How would you rate your pain intensity **right now** on a scale of 0 to 10?
*Please circle your response where **0** = **no pain** and **10** = **worst possible pain***

**No pain**	0	1	2	3	4	5	6	7	8	9	10	**Worst possible pain**

3. **In the past 2 weeks** or since the problem started, how bothersome are your low back pain symptoms? **(check one)**

☐ Not at all ☐ Slightly ☐ Moderately ☐ Very much ☐ Extremely

4. Has your low back pain spread down your leg(s) **in the last 2 weeks**?

☐ No
☐ Yes

5. Have you had pain in the shoulder or neck at some time **in the last 2 weeks**?

☐ No
☐ Yes

6. What made you decide to come to the emergency department today for your low back pain? **(check any reasons that apply)**

☐ Pain relief
☐ To find out what is wrong
☐ To get an X-ray/MRI/CT
☐ To get a referral
☐ I could not get into my family doctor
☐ To get a sick note
☐ Other (please describe):

7. When you are in pain, how frequently would you agree with the following statement: "My pain is terrible, and I think it's never going to get any better" **Please circle your response where **0** = **not at all**, and **4** = **all the time.***

**Not at all**	0	1	2	3	4	**All the time**

8. Before this current back pain episode, have you previously experienced low back pain that has lasted at least 24 hours?

☐ No **If no, skip to question 9.**
☐ Yes

b. Is your current back pain episode more severe than is usual for you?

☐ No
☐ Yes

c. Have you ever had a low back operation?

☐ No
☐ Yes

9. How confident are you that your back pain will be completely gone or much better in 3 months? **Please circle your response where **0 = not confident** and **10 = very confident***

**Not confident**	**0**	**1**	**2**	**3**	**4**	**5**	**6**	**7**	**8**	**9**	**10**	**Very confident**

10. Below are some descriptions of how some people's back pain can change over time, with pictures to show how their pain might go up or down. Please look at these and check the box next to the one option that you think comes closest to how your pain has been over the last year.

a)	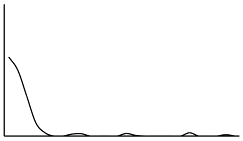	A single episode with no other major episodes of back pain	☐
b)	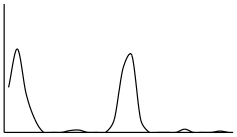	A few episodes of back pain, with mostly pain-free periods in between	☐
c)	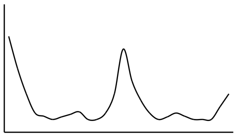	Some back pain most of the time, and a few episodes of severe pain	☐
d)	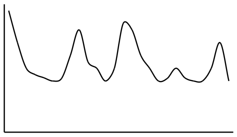	Pain that goes up and down all the time, with episodes of severe back pain	☐
e)	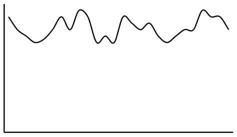	Severe back pain all or nearly all of the time	☐
f)	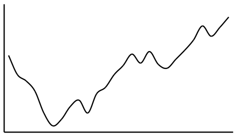	Back pain that has got gradually worse	☐
g)	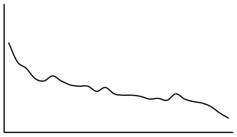	Back pain that has improved gradually	☐
h)	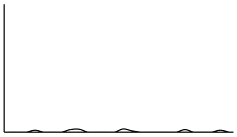	No back pain, or only the odd day with mild pain	☐

Activities and function

11. Are you: (check one)

☐ Employed full-time
☐ Employed part-time
☐ Unemployed
☐ A student
☐ Retired
☐ Unable to work due to disability
☐ Other; please specify: ____

12. In the past 2 weeks, how often have you been bothered by the following problems:

**Check one box per statement**	Not at all	Several days	More than half the days	Nearly every day
a. Little interest or pleasure in doing things.	☐	☐	☐	☐
b. Feeling down, depressed or hopeless.	☐	☐	☐	☐
c. Feeling nervous, anxious or on edge	☐	☐	☐	☐
d. Not being able to stop or control worrying	☐	☐	☐	☐

13. How many **days a week** do you do 30 minutes or more of physical activity? The activity should be enough to quicken your breathing, this should not include housework or physical activity that may be part of your job.

# Days: ______________________________________

14. On a typical weekday, how much time do you spend sitting? This may include time spent sitting at a desk, visiting friends, reading, or sitting or lying down to watch television.

Hours per day: ________ **Minutes per day:** _________ ☐ I don't know/Not sure

15. Do you agree with the following statement?
"It's really not safe for a person with a condition like mine to be physically active"

☐ No
☐ Yes

16. How confident are you in the following statements?
*Please circle your response where **0 = not at all confident** and **6 = completely confident:***

a. I can do some form of work, despite the pain ("work" includes housework, paid and unpaid work)

**Not confident**	0	1	2	3	4	5	6	**Completely confident**

b. I can live a normal lifestyle, despite the pain

**Not confident**	0	1	2	3	4	5	6	**Completely confident**

17. When your back hurts, you may find it difficult to do some of the things you normally do. This list contains some sentences that people have used to describe themselves when they have back pain. When you read them, you may find that some get your attention because they describe your situation today. Read the list, and place a check next to the sentence if you are sure that it describes you today. Remember, checkmark the sentence only if you are sure that it **describes you today**.

- ☐ I stay at home most of the day because of my back pain.
- ☐ I change my position frequently to allow my back to be more comfortable.
- ☐ I walk slower than usual because of my back pain.
- ☐ Because of my back pain, I am not doing any of the jobs that I usually do around the house.
- ☐ Because of my back pain, I use a handrail to get upstairs.
- ☐ Because of my back pain, I lie down to rest more often than usual.
- ☐ Because of my back pain, I have to hold on to something to get out of an armchair.
- ☐ Because of my back pain, I ask other people to do things for me.
- ☐ I get dressed slower than usual because of my back pain.
- ☐ I stand up only for short periods of time because of my back pain.
- ☐ Because of my back pain, I try not to bend over or kneel down.
- ☐ I find it difficult to get out of a straight-backed chair because of my back pain.
- ☐ My back is painful most of the day.
- ☐ I find it difficult to turn over in bed because of my back pain.
- ☐ Because of my back pain, my appetite is not very good.
- ☐ I have trouble putting on my socks (or stockings) because of my back pain.
- ☐ Because of my back pain, I walk only short distances.
- ☐ I sleep less than usual because of my back pain.
- ☐ Because of my back pain, I get dressed with help from someone else.
- ☐ I spend most of the day sitting because of my back pain.
- ☐ I avoid heavy jobs around the house because of my back pain.
- ☐ Because of my back pain, I am more irritable and bad tempered than usual with people.
- ☐ Because of my back pain, I go upstairs slower than usual.
- ☐ I stay in bed most of the day because of my back pain.

Other participant information

18. Have you tried any treatments for your **current episode** of back pain? **(check all that apply)**

- ☐ Physiotherapy
- ☐ Massage
- ☐ Chiropractic
- ☐ Acupuncture
- ☐ Surgery
- ☐ Other; **please specify:** ____________________
- ☐ I have not tried any treatment for my current episode of back pain

19. What type of medications have you tried for your **current episode** of back pain?

- ☐ Pain medication; **please specify:** ____________________
- ☐ Muscle relaxant; **please specify:** ____________________
- ☐ I have not taken any medication for my current episode of back pain

20. In general, how would you rate your health? **(check one)**

- ☐ Poor
- ☐ Fair
- ☐ Good
- ☐ Very good
- ☐ Excellent

21. **a.** Current height: _______________ cm OR inches (please circle units)

b. Current weight: ____________lbs OR kg (please circle units)

22. Do you currently smoke?

- ☐ No
- ☐ Yes If yes, how many cigarettes per day? ___________ How many years have you smoked? ___________

23. Highest level of education completed: **(check one)**

- ☐ < High school
- ☐ High school
- ☐ College/undergraduate
- ☐ Graduate degree

23. Current living arrangement: **(check one)**

- ☐ Living alone
- ☐ With someone else
- ☐ In a care facility
- ☐ Other; please specify: ____________

Thank you for completing this survey!
