# Supplementary material for: Development of a clinical decision support tool for diagnostic imaging use in patients with low back pain: a study protocol
Source: Diagn Progn Res. 2019 Jan 14;3:1. doi: 10.1186/s41512-019-0047-8 (PMC6460553; doi:10.1186/s41512-019-0047-8)
Supplement: Supplementary file 3 — ICD codes to identify emergent-cause low back pain. (PDF 14 kb) [file 41512_2019_47_MOESM3_ESM.pdf]

Appendix 3: ICD codes* to identify emergent-cause low back pain.

**Vertebral fracture**	**Cancer**
805.4 Compression fracture lumbar spine	162 Lung ca
805.6 Fracture coccyx/sacrum	174.9 Breast ca
809.0 Other fracture spine/trunk	183.0 Ovary ca
959.8 Multiple trauma	185 Prostate ca
S32.0 Fx lumbar vert, closed	189.0 Kidney ca
S32.1 Fx sacrum closed	191.9 Brain ca
S32.20 Fx sacrum, closed	199.1 Metastatic cancer
S32.80 Fx pelvis, closed	202.80 Lymphoma
	203.0 Multiple myeloma
**Spinal infection**	204.1 Chronic lymphocytic leukemia
041.9 Other bacterial	208.0 Acute leukemia
136.9 Infection not classified	214.9 Lipoma
324.1 Abscess, spinal	239.6 CNS mass
682.2 Abscess back	C18.9 Neoplasm of colon
G06.1 Intraspinal abscess and granuloma	C34.9 Neoplasm of lung
M46.4 Discitis	C50.9 Neoplasm of breast
	C76.7 Neoplasm, other
**Cauda equina syndrome**	C90.0 Multiple myeloma
334.60/g83.4	D36.9 Benign tumour

*Anticipated ICD codes; list to be confirmed through consultation with relevant clinicians and advisory group.
